# Supplementary material for: Long-term changes in soil biological activity and other properties of raised beds in Longan orchards
Source: PeerJ. 2024 Nov 6;12:e18396. doi: 10.7717/peerj.18396 (PMC11639206; doi:10.7717/peerj.18396)
Supplement: Supplemental Information 2 [file peerj-12-18396-s002.docx]

**Supporting Information**

**Long-term changes in soil biological activity and other properties of raised beds in Longan orchards**

Nghia Khoi Nguyen^1*^, Phuong Minh Nguyen^1^, Thy Anh Thi Chau^1^, Luan Thanh Do^1^, Ha Thu Thi Nguyen^1^, Duong Hai Vo Tran^2^, Xa Thi Le^3^, Javad Robatjazi^4^, Hendra Gonsalve W Lasar^4^, Lois Wright Morton^5^, M. Scott Demyan^6^, Huu-Tuan Tran^7^, Hüseyin Barış Tecimen^8^

^1^ Department of Soil Science, Can Tho University, Can Tho, Can Tho, Vietnam

^2^ Department of Agriculture and Aquaculture, Bac Lieu Technical and Economic College, Bac Lieu, Vietnam

^3^ School of Education, Soc Trang Community College, Soc Trang province, Vietnam

^4^ Department of Soil and Crop Sciences, Texas A&M University, College Station, Texas, United States

^5^ Department of Sociology and Criminal Justice, Iowa State University, Ames, Iowa, United States

^6^ School of Environment and Natural Resources, The Ohio State University, Columbus, Ohio, United States

^7^ Laboratory of Ecology and Environmental Management, Science and Technology Advanced Institute, Van Lang University, Ho Chi Minh, Viet Nam

^8^ Department of Environmental Sciences, College of Agriculture, Tennessee State University, Nashville, USA

*Corresponding Author:

Nghia Khoi Nguyen

Department of Soil Science, College of Agriculture, Can Tho University

Address: Campus 2, 3/2 Street, Xuan Khanh Ward, Ninh Kieu District, Can Tho City, Vietnam.

Email address: [nknghia@ctu.edu.vn](mailto:nknghia@ctu.edu.vn)

**Caption of Supplementary Table**

**Table S1**

Table S1 Information from 20 orchards collected samples in An Binh commune, Long Ho district, Vinh Long province, Vietnam.

**Table S2**

Table S2 Correlation between raised bed ages and soil physical properties of different Longan orchard soils in Vinh Long, Vietnam.

**Table S3**

Table S3 Correlation between raised bed age and chemical parameters of different Longan orchard soils in the Mekong River Delta of Vietnam.

**Table S4**

Table S4 Correlation between raised bed age and soil enzyme activities of different Longan orchard soils in the Mekong River Delta of Vietnam.

**Table S1** Information from 20 orchards collected samples in An Binh commune, Long Ho district, Vinh Long province, Vietnam.

| **Sample Code** | **Raised bed age**  **(years)** | **Longan tree age**  **(years)** | **Farm area**  **(m^2^)** |
| --- | --- | --- | --- |
| L1 | 15 | 15 | 2500 |
| L2 | 20 | 20 | 2700 |
| L3 | 20 | 20 | 3000 |
| L4 | 20 | 20 | 2000 |
| L5 | 25 | 20 | 7000 |
| L6 | 30 | 15 | 7000 |
| L7 | 30 | 30 | 4000 |
| L8 | 30 | 20 | 4500 |
| L9 | 30 | 30 | 3000 |
| L10 | 30 | 25 | 1500 |
| L11 | 38 | 30 | 2000 |
| L12 | 40 | 15 | 3000 |
| L13 | 43 | 15 | 9000 |
| L14 | 45 | 22 | 10000 |
| L15 | 45 | 15 | 1000 |
| L16 | 50 | 30 | 5000 |
| L17 | 50 | 15 | 22000 |
| L18 | 43 | 53 | 4000 |
| L19 | 55 | 17 | 6000 |
| L20 | 60 | 15 | 1500 |

**Table S2** Correlation between raised bed ages and soil physical properties of different Longan orchard soils in Vinh Long, Vietnam.

|  | **Moisture content (%)** | **Bulk density**  **(g cm ^-3^)** | **Soil porosity**  **(%)** |
| --- | --- | --- | --- |
| **Raised bed age** | -0.570^**^ | 0.666^**^ | -0.666^**^ |

*Note: ** indicate the correlation coefficients and represent significant differences at p < 0.01, respectively; n =5.*

**Table S3** Correlation between raised bed age and chemical parameters of different Longan orchard soils in the Mekong River Delta of Vietnam.

|  | **Soil organic matter (%)** | **N total (%)** |
| --- | --- | --- |
| **Raised bed age** | -0.579^**^ | -0.473^*^ |

*Note: * and ** indicate the correlation coefficients and represent significant differences at p < 0.05 and p < 0.01, respectively.*

**Table S4** Correlation between raised bed age and soil enzyme activities of different Longan orchard soils in the Mekong River Delta of Vietnam.

|  | **β-Glucosidase**  **(µg g^-1^ h^-1^)** | **Urease**  **(µg g^-1^ h^-1^)** | **Phosphomonoesterase**  **(µg g^-1^ h^-1^)** | **Phytase**  **(µg g^-1^ h^-1^)** |
| --- | --- | --- | --- | --- |
| **Raised bed age** | -0.452^*^ | -0.528^*^ | -0.487^*^ | -0.467^*^ |

*Note: * indicates the correlation coefficients and represent significant differences at p < 0.05, respectively.*
